# A general approach for retrosynthetic molecular core analysis

**DOI:** 10.1186/s13321-019-0380-5

**Published:** 2019-09-24

**Authors:** J. Jesús Naveja, B. Angélica Pilón-Jiménez, Jürgen Bajorath, José L. Medina-Franco

**Affiliations:** 1 PECEM, School of Medicine, Universidad Nacional Autónoma de México, Avenida Universidad 3000, 04510 Mexico City, Mexico; 2 Department of Pharmacy, School of Chemistry, Universidad Nacional Autónoma de México, Avenida Universidad 3000, 04510 Mexico City, Mexico; 3 Department of Life Science Informatics, B-IT, LIMES Program Unit Chemical Biology and Medicinal Chemistry, Rheinische Friedrich-Wilhelms-Universität, Endenicher Allee 19c, 53115 Bonn, Germany

**Keywords:** Analog series-based scaffold, Analog searching, Core structure–property relationships (CSPR), RECAP, Scaffold, Virtual screening

## Abstract

Scaffold analysis of compound data sets has reemerged as a chemically interpretable alternative to machine learning for chemical space and structure–activity relationships analysis. In this context, analog series-based scaffolds (ASBS) are synthetically relevant core structures that represent individual series of analogs. As an extension to ASBS, we herein introduce the development of a general conceptual framework that considers all putative cores of molecules in a compound data set, thus softening the often applied “single molecule–single scaffold” correspondence. A putative core is here defined as any substructure of a molecule complying with two basic rules: (a) the size of the core is a significant proportion of the whole molecule size and (b) the substructure can be reached from the original molecule through a succession of retrosynthesis rules. Thereafter, a bipartite network consisting of molecules and cores can be constructed for a database of chemical structures. Compounds linked to the same cores are considered analogs. We present case studies illustrating the potential of the general framework. The applications range from inter- and intra-core diversity analysis of compound data sets, structure–property relationships, and identification of analog series and ASBS. The molecule–core network herein presented is a general methodology with multiple applications in scaffold analysis. New statistical methods are envisioned that will be able to draw quantitative conclusions from these data. The code to use the method presented in this work is freely available as an additional file. Follow-up applications include analog searching and core structure–property relationships analyses.
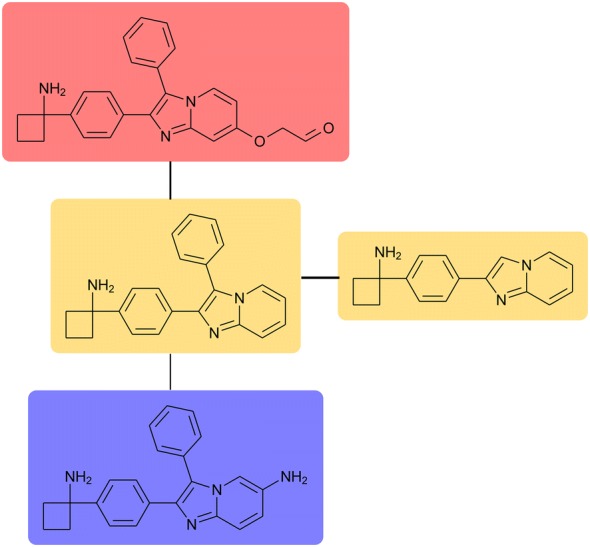

## Introduction

A general trend in drug discovery through big data is emerging [1]. In this context, many exploratory analyses for finding correlations between chemical data and biological activity have been applied, often with satisfactory results [2]. Nonetheless, many of such models require numerical molecule representations in vectors, as opposed to the complex information enclosed in a chemical structure [3]. Chemical fingerprints, a widely applied representation for converting chemical structures into information vectors, produce a result even when processing complex structures [4]. It is common that such methods detect chemical similarity between molecules even when a synthetic chemist would struggle to find substantial structure commonalities [5].

**Fig. 1:**
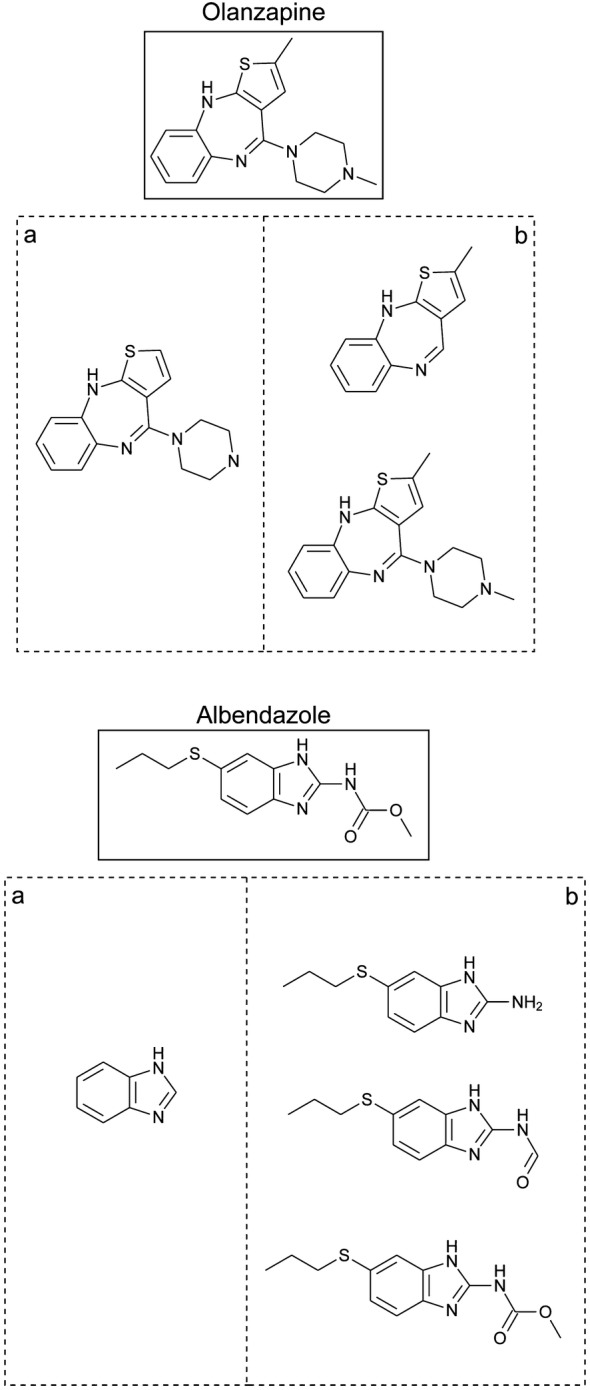
Two scaffolds definitions are applied to two exemplary molecules (olanzapine and albendazole). **a** Bemis–Murcko scaffold; **b** putative cores

In contrast to structural fingerprints, molecular scaffolds (and sub-structure methods in general) are alternative representations intuitively interpretable by a chemist, and scaffold analysis is a more chemically conservative approach than a computational prediction of structural resemblance [5]. Several approaches have been proposed to define and generate scaffolds in a consistent manner [6–8]. One of the earliest and still most common scaffold concepts was proposed by Bemis and Murcko [9] and is exemplified in Fig. 1. Section “a” of this figure shows the Bemis and Murcko scaffolds for olanzapine and albendazole. Interestingly, this scaffold concept has evolved. For instance, hierarchies of scaffolds have been proposed, which allow to associate scaffolds sharing rings and provide better clustering opportunities than classical scaffold definitions [10–12]. A more comprehensive review on scaffold analysis can be found in [8].

However, these and other classic definitions of scaffolds consider only ring systems, a rather inconvenient feature since it is not uncommon that small rings are conceptualized as side chains or part of substituents by synthetic chemists. Considering the limitations of classical scaffolds, Bajorath et al. developed a novel scaffold concept: the analog series-based scaffold (ASBS) [13] illustrated in section “b” of Fig. 1. In general, ASBS are found through a process that incorporates retrosynthetic information and restrictions in the core/molecule size ratio, thus allowing the identification of chemical analogs that can be summarized in meaningful R-group tables [14, 15]. Hence, ASBS leverage the chemical synthesis and biological relevance of scaffolds [16]. A shortcoming of the current implementation of ASBS is that it depends on the specific dataset [6]. We show below that this is a direct consequence of following the “single molecule–single scaffold” paradigm during the ASBS generation. When using ASBS for analyzing scaffold diversity or comparing scaffolds found in different datasets, it should be taken into consideration that ASBS are by design dataset-dependent.

The goal of this work is to show how softening the “single molecule–single scaffold” paradigm can lead to consistent core results that can extend the ASBS to core diversity analysis and core-property relationships analysis. Furthermore, original ASBS can be obtained on the basis of the generalized approach. Building upon the ASBS approach, we propose a conservative yet flexible general framework able to obtain synthetically relevant cores from chemical libraries, allowing applications such as analog searching through the matching of shared cores, diversity, and structure–property relationship (SPR) analyses.

This Methodology paper is organized into two major sections. First, we describe the general approach for constructing molecule–core networks. In the second section, we introduce the application of the method using two case studies, namely: core overlap analysis of two natural products datasets and core structure–activity relationship (CSAR) analysis of an analog series of Akt2 inhibitors. Perspectives for the methodology include, for example, chemical core diversity analysis, advanced SPR, and chemical analog searching. The approach has been used already for the identification of analog series and corresponding scaffolds [15].

## Methods

### Core definition

For any given molecule, a putative core is defined by two criteria [13], herein termed relevance and synthetic feasibility, further clarified as follows:

1. The relative size of the core as compared to the whole molecule is significant (relevance criterion), and
2. The core is either the whole molecule or a substructure obtained from the original molecule through a series of predefined retrosynthetic steps (synthetic feasibility criterion).

These two criteria ultimately require the user’s input to be further specified. Regarding the first criterion, previous determinations of ASBS have considered a 2:1 ratio of the scaffold vs. all substituents’ atoms [13]. The second criterion requires predefining sets of retrosynthesis rules, such as the widely used RECAP rules [17]. A user may implement other sets of available rules [18] or proprietary retrosynthetic schemes.

Importantly, given the newly proposed framework, the “single molecule–single core” paradigm underlying various scaffold definitions is no longer compulsory. On the contrary, all substructures of a molecule complying with the two criteria above are considered as putative cores, illustrated in Fig. 1b for an exemplary molecule. Our approach is able to include cyclic substructures in both cores and substituents.

A direct consequence of computing putative cores for one or more datasets of molecules is analyzing the core structures in light of scaffold criteria. Major differences compared to the scaffold concept by Bemis and Murcko (Fig. 1), are presented in Table 1.

**Table 1:** Comparison of the Bemis–Murcko scaffold and the core framework proposed in this work

Feature	Bemis–Murcko scaffold	Core framework
Number of cores per molecule	0 or 1	1 or more
Rings can be substituents	No	Yes
Considers retrosynthesis rules	No	Yes
The core is a major component of the molecule	Yes/no	Yes

### Molecule–core network

**Fig. 2:**
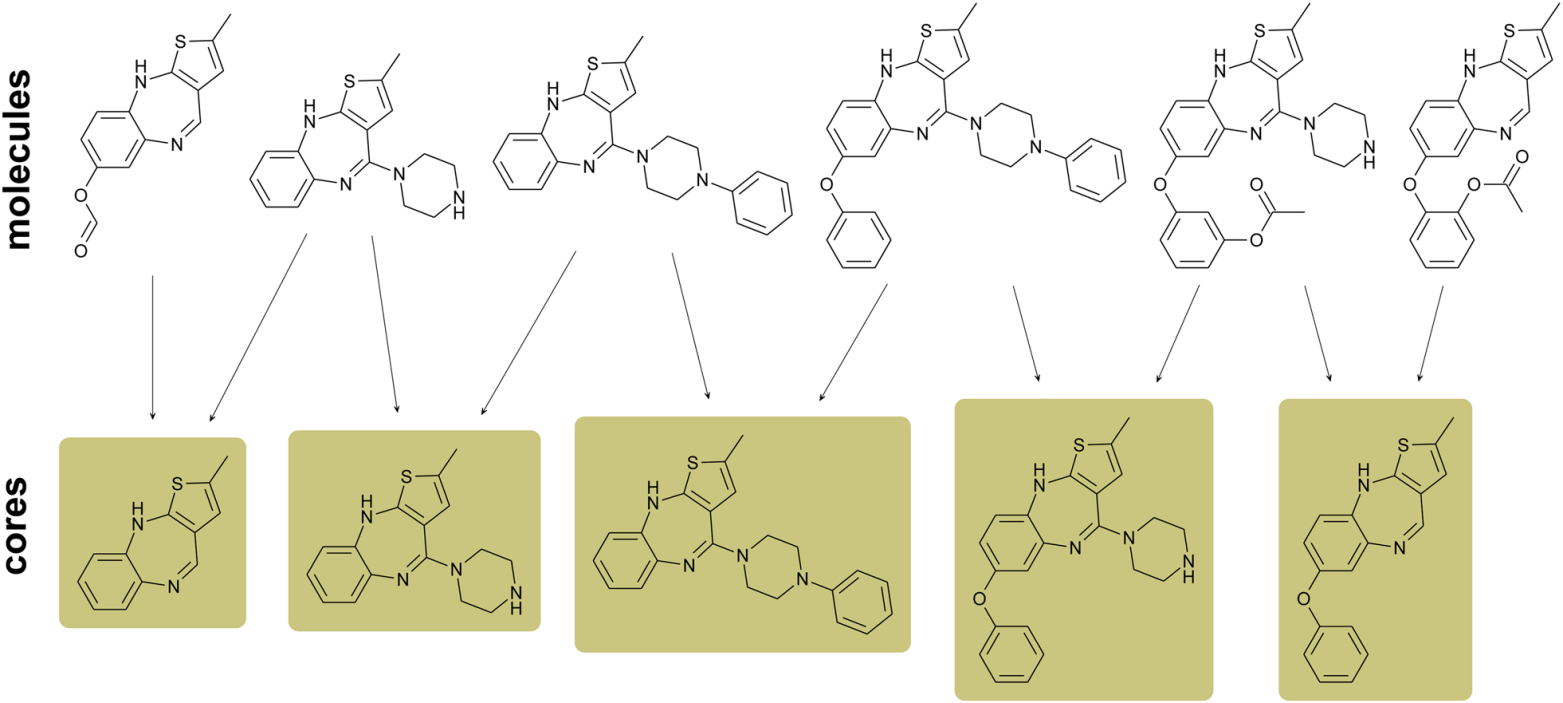
Construction of a core–molecule network for an exemplary dataset. Each molecule is connected to all of its putative cores. Thus, series can be formed if at least two molecules share a core. Note that not all molecules in a series need be pairwise analogs of each other, but a sequence of analogs must exist. For this example, only putative cores mapping to more than a single molecule are included

If the core definition described above is applied to a set of compounds, a bipartite network *G* = (*U*, *V*, *E*) can be drawn, where *U* is the set of molecules, *V* the set of putative cores, and *E* the set of edges linking molecules to their putative cores. By definition, if two molecules *u*_*1*_, *u*_*2*_ ∈ *U* can be mapped to the same *v*_*1*_ ∈ *V*, they are considered analogs. An example of a core network is illustrated in Fig. 2, where a set of six exemplary molecules is mapped to all possible cores. Separate clusters represent series. If all compounds in a series can be mapped to a single core, then the series is an analog series, and the comprehensive core is its ASBS. It has been shown that not all sets of related compounds form analog series applying this formalism since in some cases, no single core represents all compounds [15]. Moreover, to a pre-defined analog series represented by a single core, new molecules might be difficult to add. On the contrary, the use of expandable series with multiple cores makes it easy to include new compounds, which need only to be decomposed according to the same criteria and incorporated into the network. This is a consequence of accounting for all possible molecule–core relationships.

### Computational implementation

**Fig. 3:**
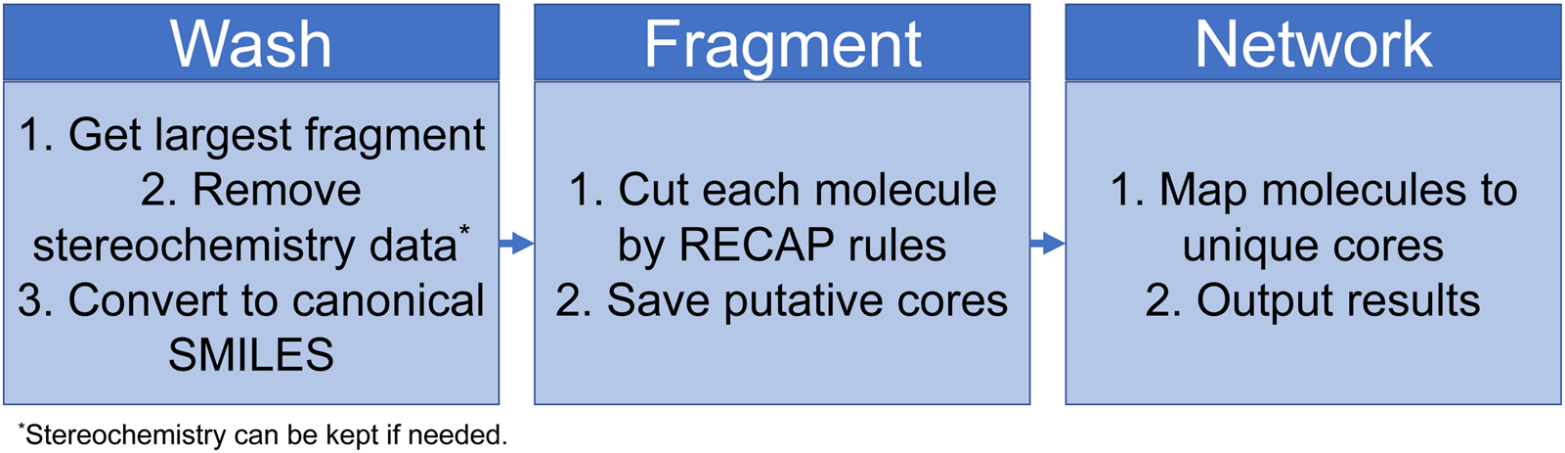
Algorithm steps for the generation of core–molecule associations

An RDKit—Python [19] implementation of the algorithm is made available in Additional files 1, 2 (see also section Availability of data and materials). The algorithm flow is depicted in Fig. 3. The code is fully parallelized and runs mostly off-memory, which means it can be used to process large chemical libraries. The input is a file with molecular structures represented as SMILES strings as well as an identifier. A “washing” script was added to remove salts, retain the largest molecular component, generate canonical SMILES, and omit stereochemistry information by default. However, stereochemistry can be retained by modifying the data preparation script. Canonical SMILES are annotated with an identifier (WID). Then, each molecule is fragmented independently, and only fragments complying with the core definition (see “Methods”) are saved. Unique cores are annotated with another identifier (MID). Finally, through network analysis, analog series are identified as disjoint subgraphs (clusters). The output is: (1) a file containing molecule–core associations (suffix: “cores.tsv”); (2) a file containing analog series–molecule associations (suffix: “ASW.tsv”); (3) a file containing analog series–cores associations (suffix: “ASM.tsv”).

## Results

The newly introduced framework has a number of potential applications such as structural analysis of compound databases including structural diversity analysis (based on the new cores), structure–property(–activity) relationships (SP(A)R), and virtual screening [12]). In this section of the Methodology paper, we discuss selected applications of the core framework.

### Core content analysis

#### Exemplary core overlap analysis in natural product data sets

To illustrate a core overlap analysis we present an example using two publicly available natural product datasets including NuBBE_DB_ [20] and BIOFACQUIM [21], which contain information about Brazilian and Mexican natural products, respectively.

**Fig. 4:**
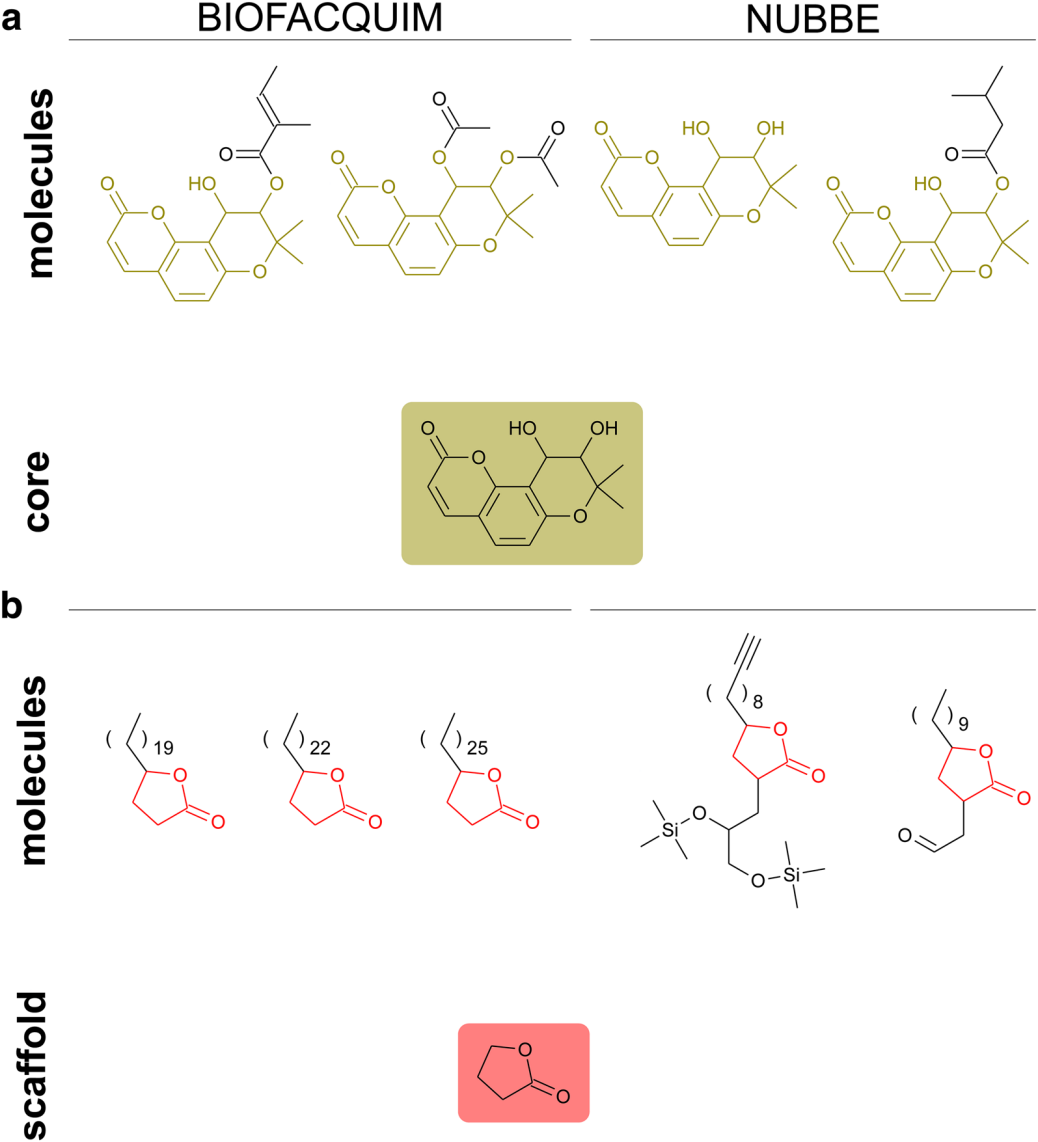
Exemplary overlapping cores and scaffolds from two datasets. **a** For any overlapping core, an analog series can be found with the core itself as its ASBS; **b** This is not necessarily the case for overlapping Bemis–Murcko scaffolds

The motivation of pursuing a scaffold overlap analysis would be to identify common and unique chemotypes in these databases. As shown in Table 2, NuBBE_DB_ and BIOFACQUIM share 49 (~ 5%) Bemis–Murcko scaffolds and around 106 (~ 1%) cores. By design, the number of unique Bemis–Murcko scaffolds can only be as high as the total number of unique molecules, while this is the minimum number of cores that can be found. This explains why more cores than Bemis–Murcko scaffolds are found. Remarkably, if a core is shared between two databases, an analog series might be constructed for that core (Fig. 4a). On the other hand, a shared Bemis–Murcko scaffold, which does not consider the core-to-substituents ratio by design, might not represent a meaningful analog series (Fig. 4b).

**Table 2:** Core and Bemis–Murcko scaffold overlap of NuBBE_DB_ vs BIOFACQUIM databases

	Measurement	BIOFACQUIM	NuBBE_DB_	Both
	Unique molecules intraDB	399	2018	2417
	Unique molecules interDB	344	1963	2362 (55 shared)
Cores	Cores intraDB	1356	15,758	17,114
	Unique cores intraDB	1153	11,738	12,289
	Unique cores interDB	1047	11,632	12,785 (106 shared)
Bemis–Murcko scaffolds	Scaffolds intraDB	396	1921	2317
	Unique scaffolds intraDB	176	754	930
	Unique scaffolds interDB	127	705	881 (49 shared)

Similar overlap analysis can be performed with other larger natural product databases such as the Dictionary of Natural Products [22], the Universal Natural Product Data Set [23] or basically any other compound collection. Here, we illustrate the method with two natural product datasets as examples. Of note, quantitative diversity metrics remain to be developed, similar to those available to quantify scaffold diversity based on Bemis–Murcko scaffolds [24].

### Core structure–property (activity) relationship analysis: “hit-to-lead cores”

**Fig. 5:**
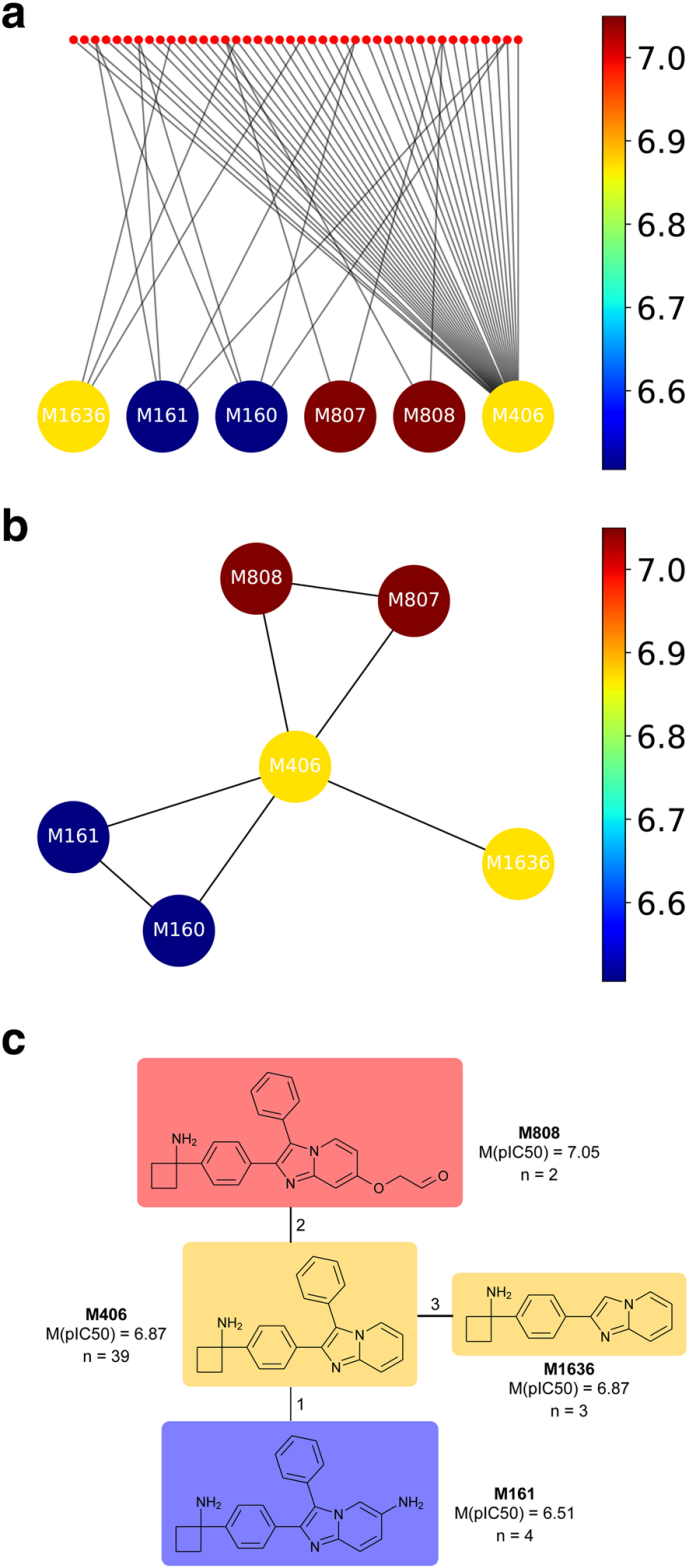
Core structure–activity relationship visualization for the largest series in a dataset of Akt2 inhibitors. **a** Molecule–core bipartite network. Molecules are shown as small red dots, while cores are represented as larger dots and colored by the median of the pIC_50_ of the molecules represented by them. **b** Core network obtained from the molecules-cores bipartite network. Nodes are putative cores and edges are drawn between nodes that share at least one compound in the dataset; **c** final CSAR visualization. Redundant cores were omitted and chemical structures were added to the core’s network

Substructure and scaffold-based representations are commonly used in many areas of chemistry. An example is R-group tables to assist in the analysis of SPRs [25, 26]. Considering cores changes the view of SPR analysis. For instance, every collection of molecules linked to a single core can be considered an analog series, for which SPR can be conducted using an R-group table. Moreover, molecules can be assigned to more than a single core. Therefore, the progression of an analog series can be readily visualized from the core perspective (Fig. 5). Analyzing a database and identifying the most relevant analog series with a given activity, can be considered “lead discovery”. Such an approach prioritizes activity of the analog series over its size measured in the number of analogs it contains. This can be accomplished best by considering the properties in the whole molecule–core network and then selecting enriched cores. Such cores will represent an analog series where the desired property tends to appear, plus different decorations on the scaffold retain the property. Therefore, these cores could be considered leads for drug discovery programs. We call these cores “hit-to-lead cores”, as they can also resemble a hit in the sense that it can be found from exploratory and high-throughput drug discovery campaigns.

#### Exemplary CSAR analysis

Herein, we illustrate the application of CSAR analysis with a dataset of Akt2 inhibitors extracted from ChEMBL 24 [27, 28]. For preprocessing of the data, only compounds with reported IC_50_ values and standard type “=” were considered. Furthermore, duplicates were removed and the maximum ChEMBL activity values were kept. The dataset was first run through the *cores.py* script (see Additional files 1, 2) and the output was used for CSAR analysis. A Jupyter Notebook with the CSAR analysis is provided as an Additional files 1, 2 as well.

79 series had at least two compounds, and 24 series had at least five. The largest series contained 42 compounds. We analyzed the SAR of this largest series and found that only six cores were connected to more than a single compound. As shown in Fig. 5a, a bipartite network is constructed, where one part of the network is the molecules and the other their putative cores. Edges map molecules to their putative cores. In this way, for any given property, a statistical distribution can be obtained for each core through analogs mapping to the core. Also, the bipartite network allows examining the relevance of the cores. In the example shown in Fig. 5a, the core labeled **M406** represents a larger subset of molecules (represented by red dots at the top of the figure). Note that the cores labeled **M807**, **M808**, **M160**, and **M161** are mapped to the same subset of molecules (Fig. 5a).

The molecule–core bipartite network can be condensed to a core network representation. Figure 5b illustrates a molecule–core network taken the information from Fig. 5a. The network shows the relationship of the core labeled **M406** with five other cores. An edge between two cores means that they share at least one molecule. As in Fig. 5a, the dots in Fig. 5b are colored by the median of the pIC_50_ of the associated molecules using a continuous color scale. The core network shows that three subregions in the CSAR can be found. Furthermore, in this case, there is a gradient, where the most active cores (**M807** and **M808**) are connected to cores with medium activity (**M406**) but not to those with low activity (**M160** and **M161**).

Figure 5c shows a more detailed CSAR visualization for this series in Fig. 5a, adding the chemical structures to the core’s network and removing redundant cores by keeping only the largest. In this example, Fig. 5c indicates that the four Akt2 inhibitors sharing the core **M161** with an amine substitution in the imidazopyridine ring (average pIC_50_ = 6.51) are less active than the two molecules having the related core **M808** but with a substituent with negative partial charges (average pIC_50_ = 7.05).

### Identification of analog series and corresponding scaffolds

In a recent publication, a direct application of the core framework for finding ASBS was introduced [15]. By definition, analog series must have a common scaffold and be disjoint from each other according to the paradigm of “single molecule–single scaffold” paradigm. To this end, the initial bipartite network of molecules and their putative cores can be used as a starting point. Then, the number of putative cores has to be reduced to the minimum, and subnetworks are not allowed to overlap. This can be achieved by an iterative greedy selection of cores according to which cores that are more represented in the dataset persist and disqualify secondary cores.

## Discussion

Scaffold content and diversity analysis are common practice to explore the chemical space of compound data sets and perform classifications based on a structure representation that is highly intuitive [29–31]. There are multiple ways of defining chemical scaffolds or cores (see [32] for a comprehensive review). Of note, hierarchical scaffolds might allow each molecule to have more than a single scaffold. Nevertheless, the level a scaffold occupies in the hierarchy is arbitrary and depends on the dataset. In our general core approach, core structures are followed horizontally, rather than following a hierarchy, as they progress (see Fig. 2). A further issue that remains to be addressed is matching of cores with small chemical changes in rings.

Herein, we have introduced a novel framework for performing scaffold analysis, which is an extension and generalization of the ASBS approach. Several exemplary applications of the approach were presented. In contrast to the generation of ASBS, where the main objective is representing analog series in a given dataset, our approach avoids any possible information loss as a consequence of not considering all possible molecule–core relationships. In consequence, the new approach generates and stores more data than required for ASBS, but this ensures consistency and interoperability among datasets. Also, for newly generated or updated chemical libraries it is possible to extend the library of cores by only processing new molecules that were added. Only in the context of a chemical dataset, cores can be chosen that represent as many molecules as possible. Reducing the number of cores might be feasible for SPR analysis, but not for comprehensively comparing core overlap between databases.

Among the limitations of the newly presented core framework is the often increased computational cost compared to chemical fingerprint methods or conventional scaffold analysis following Bemis and Murcko. Nonetheless, the off-memory and parallel nature of the scripts make it feasible to process a database as large as ChEMBL_24 on a desktop computer in less than 24 h. Furthermore, the results depend on the definition of the retrosynthetic rules to be considered and the specific core-to-fragments ratio. We anticipate that the definition of these two parameters impacts the performance of the approach in a given project. Also, as with any approach extracting knowledge retrospectively from a dataset, data quality will obviously affect the analysis.

The method is expected to have the potential for a variety of applications. Given the scope of this Methodology paper, we present two exemplary applications in diversity and SAR analysis. Also, this new framework opens the door to new and more informative SAR visualization approaches. For instance, constellation plots have recently been proposed as a novel approach for visualizing analog series in the chemical space [33].

## Conclusions and perspectives

In this study, a new and general method inspired by the ASBS concept is introduced. Exemplary applications are shown to establish a proof-of-concept using data from medicinal and natural product chemistry. Scaffold content and diversity analysis are fundamental to characterize compound databases. The results of the recently developed definition of ASBS have proven the chemical and biological usefulness of identifying core scaffolds through retrosynthetic rules and size restrictions. Other applications include the identification of ASBS for hit identification and structure–property analysis. Using the proposed framework, new questions can be answered when comparing datasets, such as how many molecules in a dataset match a synthetic analog in another dataset, or how often cyclic substructures are found as substituents of a particular core in the context of a given dataset.

Going forward, the new core framework might be systematic to analog searching and core hopping.

## Supplementary information

**Additional file 1.** Source code for getting core data.

**Additional file 2.** A zip file containing a Jupyter Notebook with the exemplary CSAR analysis for the Akt2 dataset, as well as the data and secondary scripts required.

## Data Availability

Source code for getting core data is provided using the free RDKit Python package as an additional file. Requirements: Linux OS, an RDKit environment, packages: pandas, NetworkX, Dask. A.zip file containing a Jupyter Notebook with the exemplary CSAR analysis for the Akt2 dataset is provided as well, including the output data from the script.

## Abbreviations

ASBS: analog series-based scaffold
CSAR: core structure–activity relationship
CSPR: core structure–property relationship
RECAP: retrosynthetic combinatorial analysis procedure
SAR: structure–activity relationship
SMILES: simplified molecular-input line-entry system
SPR: structure–property relationship
