# Supplementary figures and images for: A general approach for retrosynthetic molecular core analysis
Source: J Cheminform. 2019 Sep 24;11:61. doi: 10.1186/s13321-019-0380-5 (PMC6760108; doi:10.1186/s13321-019-0380-5)

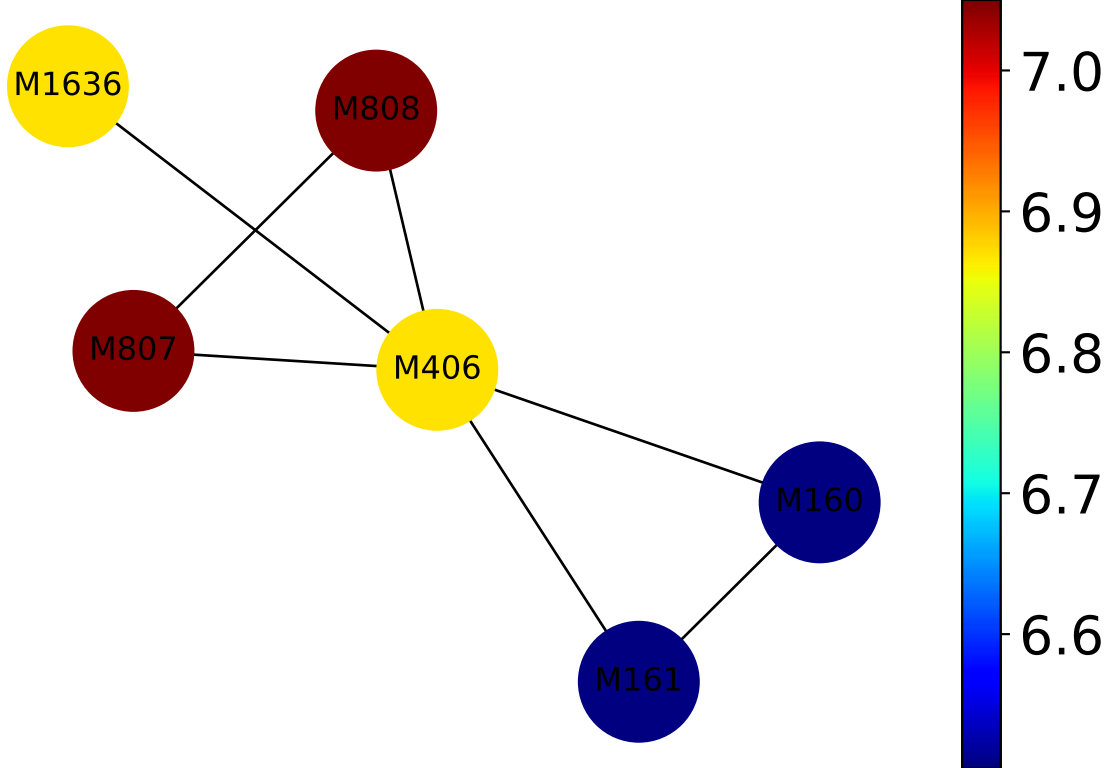

Supplement: Supplementary file 2 — Additional file 2. A zip file containing a Jupyter Notebook with the exemplary CSAR analysis for the Akt2 dataset, as well as the data and secondary scripts required. [file 13321_2019_380_MOESM2_ESM.zip › akt/cores.pdf]
